# Animal models of pituitary neoplasia

**DOI:** 10.1016/j.mce.2015.08.024

**Published:** 2016-02-05

**Authors:** K.E. Lines, M. Stevenson, R.V. Thakker

**Affiliations:** Academic Endocrine Unit, Radcliffe Department of Medicine, University of Oxford, Oxford Centre for Diabetes, Endocrinology and Metabolism (OCDEM), Churchill Hospital, Headington, Oxford OX3 7LJ, UK

**Keywords:** Pituitary, Adenoma, Carcinoma, Mouse, Rat, Multiple endocrine neoplasia type 1

## Abstract

Pituitary neoplasias can occur as part of a complex inherited disorder, or more commonly as sporadic (non-familial) disease. Studies of the molecular and genetic mechanisms causing such pituitary tumours have identified dysregulation of >35 genes, with many revealed by studies in mice, rats and zebrafish. Strategies used to generate these animal models have included gene knockout, gene knockin and transgenic over-expression, as well as chemical mutagenesis and drug induction. These animal models provide an important resource for investigation of tissue-specific tumourigenic mechanisms, and evaluations of novel therapies, illustrated by studies into multiple endocrine neoplasia type 1 (MEN1), a hereditary syndrome in which ∼30% of patients develop pituitary adenomas. This review describes animal models of pituitary neoplasia that have been generated, together with some recent advances in gene editing technologies, and an illustration of the use of the Men1 mouse as a pre clinical model for evaluating novel therapies.

## Highlights

• Over 40 animal models of pituitary neoplasia have been reported.
• Animal models have confirmed roles of genes implicated in pituitary tumourigenesis.
• Animal models have revealed novel genes involved in pituitary tumourigenesis.
• Menin is a key protein in suppressing pituitary tumour development.

## 1 Introduction

Pituitary tumours represent 10–15% of all intracranial tumours. The standardised incidence rate of pituitary tumours is ∼4/1,00,000, with the higher incidence being in females (∼5/1,00,000) (Tjornstrand et al., 2014, Raappana et al., 2010, Gittleman et al., 2014). Pituitary tumours may occur as part of hereditary syndromes (e.g. Multiple Endocrine Neoplasia Type 1 (MEN1) or Type 4 (MEN4)), or as an isolated (non-syndromic) disorder which may be inherited (e.g. Familial Isolated Pituitary Adenomas (FIPA)), or more commonly (>95%) as non-familial (i.e. sporadic) neoplasms (Yates et al., 2014). Pituitary tumours are also classified according to their hormonal production as: lactotrophinomas (secreting prolactin), that comprise ∼50% of tumours; gonadotrophinomas (secreting follicle stimulating hormone (FSH) or lutenising hormone (LH), but predominantly non-functioning), comprising ∼30% of tumours; somatotrophinomas (secreting growth hormone (GH)), comprising 15–20% of tumours; corticotrophinomas (secreting adrenocorticotropic hormone (ACTH)), comprising 5–10% of tumours; and thyrotrophinomas (secreting thyroid stimulating hormone (TSH)), comprising <1% of tumours. However, it is important to note that pituitary adenomas may secrete more than one hormone (Levy, 2008). Over 99% of pituitary tumours are benign adenomas with <0.2% being carcinomas that may metastasise (Scheithauer et al., 2006).

To date, over 30 animal models of pituitary tumourigenesis, usually mouse models, have been generated, using gene knockout and over-expression approaches. Evaluation of these models has increased our understanding of pituitary tumour biology and of the roles of oncogenic and tumour suppressor genes. This review will discuss these animal models of pituitary neoplasia, focussing on rodent models, together with the methods used in their generation. In addition, use of a *Men1* mouse model in evaluating approaches to targeted therapies will be reviewed.

## 2 Pituitary neoplasia models

Pituitary neoplasia may result from mutations involving either activation of a dominant gain-of-function oncogene, or inactivation of a recessive loss-of-function tumour suppressor gene. These mutations have been discovered by studies of pituitary tumours from patients, or from animal models generated for other disorders. To date, human studies of familial syndromes and sporadic disease have indicated the involvement of >35 genes in the development and progression of pituitary neoplasias (Table 1). Animal models harbouring mutations of ∼35% of these genes have been generated, and animal models of mutations in genes not previously implicated in pituitary neoplasia have also been generated, such that over 40 animal models of pituitary neoplasia have been generated, with the majority of these animal models being mutant mice (Table 2). Many of these models represent human syndromes e.g. MEN1 (Crabtree et al., 2001, Bertolino et al., 2003a, Biondi et al., 2002, Loffler et al., 2007a, Loffler et al., 2007b, Harding et al., 2009) and MEN4 (Kiyokawa et al., 1996, Nakayama et al., 1996, Fero et al., 1996), as well as representing a range of pituitary neoplasms that include hyperplasia, adenomas and carcinomas (Table 2). These pituitary tumours may secrete hormones such as prolactin, GH, ACTH, FSH, LH and TSH, or they may be non-secreting, which is also referred to as non-functioning adenomas (Table 2). These models have been generated using different methods, which will be briefly reviewed below.

### 2.1 Generation of animal models

Mutant animal models may be generated using: gene deletion (knockouts); over-expression by transgenic expression of wild type or mutant alleles; mutagenesis using chemicals e.g. *N*-ethyl-*N*-nitrosourea (ENU), or radiation; drugs e.g. long-term oestrogen treatment; and the breeding of animals with spontaneously arising abnormalities.

#### 2.1.1 Gene deletion models

**Fig. 1:**
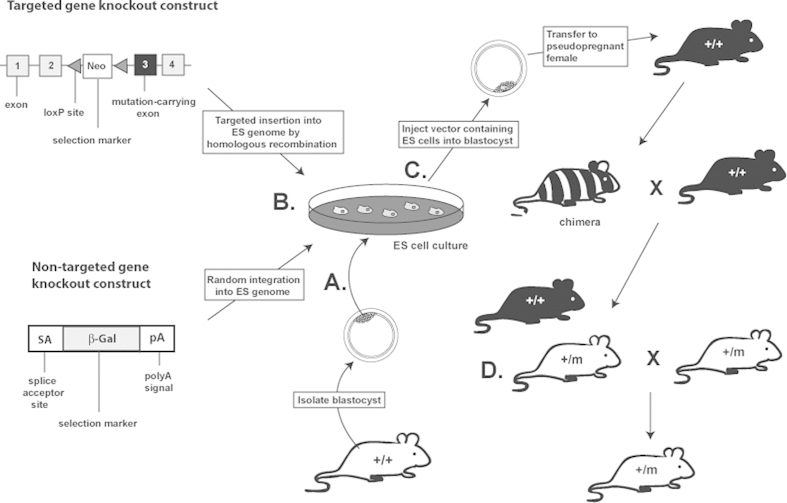
Gene knockout methods using embryonic stem (ES) cells. A Totipotent ES cells are isolated from the inner cell mass of a blastocyst from a wild type mouse and cultured. B Targeted or non-targeted vectors are introduced into the genome of the ES cells. ES cells in which homologous recombination or random integration and i.e. gene knockout, has occurred are selected using incorporated markers (e.g. Neo). C Selected ES cells are injected into a blastocyst obtained from a different strain to that in step A, and implanted into a pseudopregnant female, which is the same strain as the injected blastocyst. This results in chimeric offspring containing genetic information from both the manipulated ES cells of the original mouse strain and genetic information of the second blastocyst/pseudopregnant female of a different strain. To generate offspring heterozygous for the gene knockout, chimeric offspring are bred with wild type mice. D Heterozygous offspring can be interbred to generate homozygous knockout mice. m-mutated allele; + – wild type allele.

Gene deletion by homologous recombination (also referred to as knockout) is one of the most widely used methods to generate specific mouse models. Methods are based on modifying the gene of interest in embryonic stem (ES) cells (Fig. 1). In conventional knockout models, a vector construct comprising a plasmid or attenuated virus encoding a DNA sequence with homology to the target gene, but carrying a mutated base or bases resulting in loss of the protein, together with a positive selection marker (e.g. *Neo*), flanked by LoxP sites (allowing subsequent excision from selected cells), is introduced into ES cells where it undergoes homologous recombination resulting in stable integration of the mutated gene into the ES cell genome (Martin, 1981, Hall et al., 2009) (Fig. 1A). These modified ES cells are then injected into a mouse blastocyst to produce chimeric offspring, and ultimately mice heterozygous for the introduced mutation (Fig. 1C). These heterozygous mice may then in turn be interbred to produce homozygous mice, providing the mutation is not embryonic lethal (Fig. 1D). The option of producing either heterozygous or homozygous models can also provide further insights into whether phenotypes are inherited in a dominant or recessive manner. In tumour models this is of particular importance as often patients carry a heterozygous mutation but require a second hit to cause loss of heterozygosity (LOH), and the development of a tumour, for example in patients with MEN1 (Thakker, 2013). New strategies have been developed to generate mouse knockout models on a large scale. For example, non-targeted gene knockout models have been generated using gene trap (Collins et al., 2007a, Collins et al., 2007b) that comprises a plasmid or virus vector containing a promoter-less selectable marker (e.g. β-galactosidase) flanked by a splice acceptor site and a polyA signal, that when introduced into ES cells randomly inserts into the genome (Fig. 1B). If the vector inserts into the intron of a gene then the splice acceptor generates fusion transcripts of the selection marker with the exons upstream in the endogenous gene, thereby leading to truncation and knockout of the protein (Stanford et al., 2001). As the gene trap randomly inserts into the ES cell genome sequencing analysis can be performed to identify the site of gene trap insertion and therefore to confirm the gene knockout. More recently a targeted gene trapping method has been developed in which the vector also contains regions homologous to the gene of interest (Collins et al., 2007a, Collins et al., 2007b, Friedel et al., 2005).

**Fig. 2:**
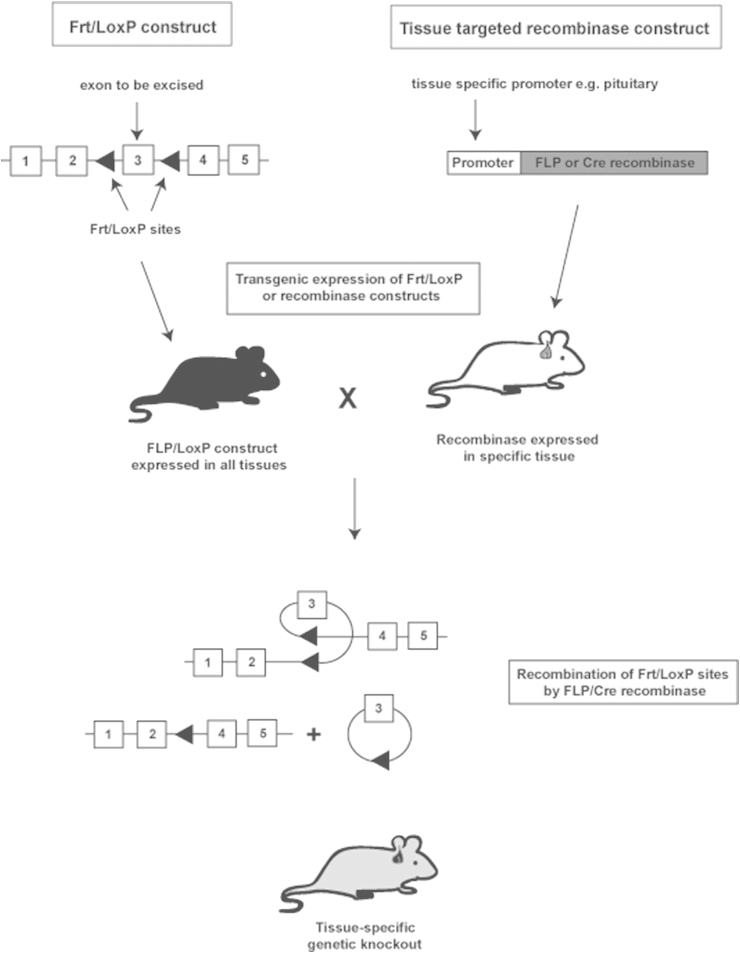
Conditional gene knockout. Gene knockout models can be generated using the FLP-Frt or Cre-LoxP systems. This requires the generation of two constructs: 1) a construct containing Frt or LoxP recognition sites inserted into the intron sequences flanking the genomic region to be knocked out; and 2) a construct containing a FLP or Cre recombinase under the control of a tissue-specific promoter. These constructs are introduced into two different mouse strains using knockin/transgenic over-expression methods, to generate one mouse expressing the Frt/LoxP flanked genomic sequence in all tissues, and one mouse expressing FLP/Cre recombinase in a specific organ e.g. pituitary. These two mouse lines are then bred to generate a mouse containing both the Frt/LoxP flanked genomic sequence and the tissue-specific recombinase. In FLP/Cre-expressing tissues e.g. pituitary, the FLP/Cre binds to its target Frt/LoxP sites and catalyses recombination of the DNA, leading to excision of the genetic material contained between the target sites.

In conventional or gene trap knockout models, generation of homozygous mice may result in embryonic lethality, as illustrated by *Men1*^−/−^ mice (Crabtree et al., 2001, Bertolino et al., 2003a, Bertolino et al., 2003b, Loffler et al., 2007a, Loffler et al., 2007b, Harding et al., 2009, Fisher et al., 2009, Lemos et al., 2009), indicating that the encoded protein, menin, plays a pivotal role in embryonic development. This embryonic lethality can be overcome by use of conditional knockout models that are tissue or cell type specific. These models can be produced by utilising *Cre*-LoxP or FLP-*Frt* systems (Fig. 2), whereby the genomic region of interest is flanked by LoxP or flippase (FLP) recombination target (Frt) sites. These sites are recognised by Cre recombinase or FLP enzymes, respectively, which excise the DNA sequence floxed by the LoxP or Frt sites (Michael et al., 1999). This method of generation requires two mouse lines, one line containing the genomic region of interest flanked by LoxP or Frt and another line expressing the tissue-targeted Cre or FLP, which are generated by transgenic methods (see Section 2.1.2). These mice are then crossed to generate mice expressing both the flanked construct and the recombinase. Tissue targeting of the Cre or FLP is achieved by restricting their expression using a tissue-specific promoter, for example rat growth hormone releasing hormone receptor (*Ghrh* receptor) to restrict Cre expression to the pituitary (Yin et al., 2008). Inducible models that allow control over the timing of gene knockout can also be generated using fusion proteins. For example, a modified ligand-binding domain of the oestrogen receptor can be fused to Cre, which only upon administration of tamoxifen (which binds the oestrogen receptor), translocates to the nucleus and excises the floxed DNA region, allowing knockout of the gene at a chosen time point during the animals life span (Fisher et al., 2009).

#### 2.1.2 Knockin and transgenic models

Knockin and transgenic models can be generated to assess gain-of-function mutations or constitutive over-expression of genes, respectively. The targeted knockin approach is similar to the conventional knockout method described above and in Fig. 1, except that the introduced construct is designed to cause gene over-expression, for example by incorporation of a gain-of-function mutation (Hall et al., 2009, Piret and Thakker, 2011). The transgenic model differs from the knockin model in that a cDNA construct, containing cDNA of either an endogenous gene (i.e. wild type construct) or a gene of interest carrying a desired mutation, and an appropriate promoter and poly(A) sequence, is injected into the pronucleus of fertilised mouse oocyte, which is implanted into a pseudopregnant female (Haruyama et al., 2009). This transgene can randomly insert into the genome, thereby introducing additional genetic information, which upon transcription results in over-expression of the inserted wild type or mutant gene (Piret and Thakker, 2011, Gordon et al., 1980). Transgenic approaches can therefore provide a tool to examine dominant negative effects of mutant proteins, as often mutations are heterozygous and therefore patients express both mutant and wild type proteins.

#### 2.1.3 Gene editing methods

Recent advances in generating animal models involve the technique of genome editing. Gene editing can be achieved by multiple methods including the use of zinc finger nucleases and transcription activator-like effector nucleases (TALENs). The most recently developed method of genome editing, however, is that using the clustered regularly interspaced short palindromic repeats (CRISPR)-CRISPR associated (Cas) system (Seruggia and Montoliu, 2014). The CRISPR/Cas system utilises a prokaryotic adaptive immune response mechanism in which RNA transcribed from small segments of plasmids or viral genomes that have been incorporated as CRISPRs during an earlier infection, are used to target Cas9 proteins to new infections where they cleave the DNA and therefore inactivate invading plasmids or viruses (Seruggia and Montoliu, 2014). This system can be used to target Cas9 proteins to specific DNA regions by developing a synthetic targeting RNA template, to cause double strand breaks, which depending on the DNA repair mechanisms employed, allows both the generation of knockout and knockin models (Fig. 4). Mouse models can be produced by either editing an ES cell or by direct microinjection into a zygote, with the advantage that multiple genes can be targeted in parallel, making the generation of, for example double knockout mice more efficient as it negates the need for multiple breeding cohorts (Wang et al., 2013).

#### 2.1.4 Chemical or radiation induced mutagenised models

**Fig. 3:**
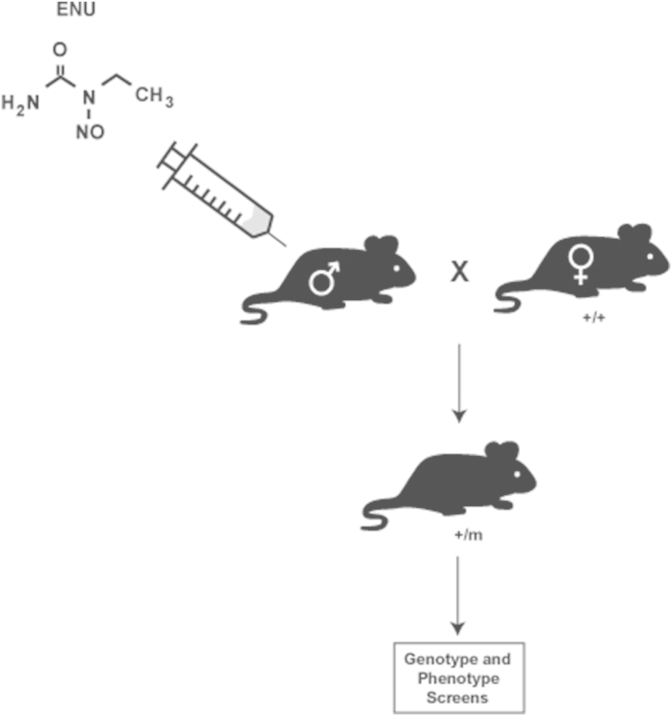
N-ethyl-N-nitrosourea (ENU) mutagenesis. ENU is a chemical mutagen that induces point mutations via transfer of its ethyl group (CH_3_) to guanine residues, which in turn causes mispairing during DNA replication. ENU is injected into male mice where it causes mispairing during spermatogenesis, and therefore induces mutations into sperm DNA. Mutagenised mice are mated with female mice of the same strain to generate offspring that have inherited the introduced mutations. Genetic and phenotypic screens are performed to characterise the mutation. m-mutated allele; +–wild type allele.

**Fig. 4:**
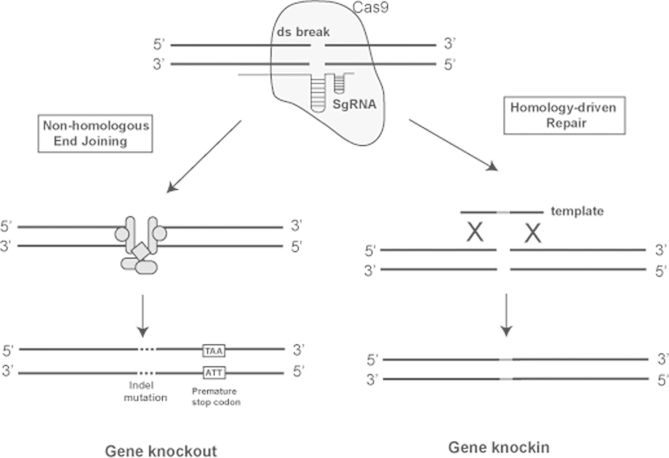
Gene editing using CRISPR/Cas. The CRIPSR/Cas system requires three components: a CRISPR-associated nuclease, for example Cas9; a single guide RNA (SgRNA) consisting of a guide sequence that binds the target DNA, a scaffold sequence for Cas9 binding and a terminating hairpin; and for gene knockin an oligo template containing the sequence to be inserted. The Cas9 is targeted to a specific genomic site by the SgRNA, where it induces a double strand break. In the absence of a repair template this break is repaired by non-homologous end joining, an error prone mechanism that leads to insertion/deletion (indel) mutations, which in turn cause frameshifts and the occurrence of premature stop codons, thereby leading to gene knockouts. If a DNA repair template is present the double strand break is repaired by a homology-driven repair mechanism, using this template, therefore genetic information can be knocked-in by including, for example, gain-of-function point mutations in the inserted template.

Chemicals and radiation can induce alterations in the DNA sequence, which can lead to either gain-of-function (hypermorphic), loss-of-function (hypomorphic) or haploinsufficiency of inherited mutations. The alkylating agent *N*-ethyl-*N*-nitrosourea (ENU) is the most potent mutagen in mice, introducing point mutations via transfer of the ENU alkyl group to DNA bases, and thereby causing mispairing during DNA replication (Piret and Thakker, 2011, Acevedo-Arozena et al., 2008). The ENU is injected into male mice to mutagenise sperm and allow the mutations to be inherited, and offspring assessed for both genetic and phenotypic differences (Fig. 3). For screening genetic differences, archives of DNA can be examined for mutations in a gene of interest (Acevedo-Arozena et al., 2008). For phenotypic screening, the offspring of the mutagenised mice are assessed using biochemical, morphological and behavioural tests and then the DNA sequenced to elucidate novel genes associated with disease (Acevedo-Arozena et al., 2008, Bentley et al., 2014).

#### 2.1.5 Spontaneously occurring mutations

Phenotypes can also occur spontaneously in animal models, for example due to either naturally occurring mutations, or viral incorporation. This is especially apparent in neoplasia models, and some strains of mice (e.g. C_3_H) and rats (e.g. Sprague–Dawley) have been reported to have a much higher tumour incidence, compared to other strains (Dusing-Swartz et al., 1979, Son and Gopinath, 2004, Rosenberg et al., 2009, Son et al., 2010, Mitsui et al., 2013, Carlus et al., 2013). Spontaneous models however are generally less predictable than those specifically generated.

#### 2.1.6 Non-genetic methods

Non-genetic methods have also been used to generate models of pituitary neoplasia. These include long-term hormone administration to induce tumour development, for example oestrogen treatment of ovariectomised rats (Mitsui et al., 2013, Wiklund et al., 1981a, Wiklund et al., 1981b) and injection of carcinogenic agents, for example cadmium (an element able to mimic oestrogen) to induce the development of multiple tumour types (Waalkes, 2003). The use of chemical carcinogens is advantageous as they provide mechanistic insights into environmental factors contributing to tumour development. There are however environmental concerns with their use, and reproducibility.

#### 2.1.7 Animal model repositories

Conventional gene knockout, gene knockin and transgenic methods have been successful in generating models of pituitary neoplasia. Many of these models are available from repositories, as live mice, for example through the European Mouse Mutant Archive (EMMA; Europe; http://strains.emmanet.org/), the Wellcome Trust Sanger Institute (UK; https://www.sanger.ac.uk/resources/mouse/) or Jackson Laboratory (USA; http://jaxmice.jax.org/). These repositories also have available mutagenised ES cell clones, to establish mouse models. Information on the models and availability of ES cells can be found from, for example, the International Mouse Phenotyping Consortium (IMPC; https://www.mousephenotype.org/). In addition, ENU mutagenised sperm and live mice are available from MRC Harwell (UK; http://www.har.mrc.ac.uk/).

### 2.2 Mouse pituitary neoplasia models

The majority (∼90%) of existing pituitary neoplasia models have been established using mice (Table 2). These models have been predominantly generated using gene knockout and over-expression methods.

#### 2.2.1 Knockout models

To date, homologous deletions of 10 different genes, which are *Men1*, *Cdkn1b*, *Prkar1a*, *Rb*, *Cdkn2b* (encoding p19), *Drd2*, *Cdkn2c* (encoding p18), *Aip*, *Prl* and *Prlr*, have been reported to yield mouse knockout models for pituitary tumours. Of these 10 genes, 5 (*Men1*, *Cdkn1b*, *Prkar1a*, *Cdkn2b* and *Aip*) are tumour suppressors that are associated with human familial disorders with pituitary tumours. Thus, *Men1*^+/−^ mice develop tumours consistent with MEN1 (Crabtree et al., 2001, Bertolino et al., 2003a, Bertolino et al., 2003b, Biondi et al., 2002, Loffler et al., 2007a, Loffler et al., 2007b, Harding et al., 2009), which is characterised by the occurrence of tumours of the parathyroid glands (in ∼95% of patients), pancreatic islets (in ∼40% of patients) and the anterior pituitary (in ∼30% of patients) (Thakker, 2013). The majority of pituitary adenomas in MEN1 patients are prolactinomas, followed by somatotrophinomas, corticotrophinomas and non-functioning adenomas (Thakker, 2013). Pituitary adenomas occurring in individuals with MEN1 are generally larger and more aggressive than sporadic tumours, and 2–3% of sporadic pituitary adenomas have *MEN1* mutations (Thakker, 2013, Verges et al., 2002, Machens et al., 2007). The *MEN1* gene is located on human chromosome 11q13 and contains 10 exons, with exon 1 non-coding. More than 1000 different germline mutations have been found in the coding region and splice sites of the *MEN1* gene, with the majority (75%) predicted to yield truncated forms and loss of its encoded protein, menin (Lemos and Thakker, 2008). The knowledge of these mutations, and that loss of menin can cause tumour development, has led to the generation of a number of *Men1* mouse models. In mice the *Men1* gene is located on chromosome 19, but has an exon-intron organisation similar to that of the human gene (i.e. 10 exons), and the mouse menin protein shows 97% homology with the human menin protein (Stewart et al., 1998). Six different mouse models for MEN1 have been generated, consisting of 4 conventional heterozygous and 2 conditional homozygous *Men1* knockouts, and 5 of these 6 models develop pituitary adenomas (Crabtree et al., 2001, Bertolino et al., 2003a, Bertolino et al., 2003b, Biondi et al., 2002, Loffler et al., 2007a, Loffler et al., 2007b, Harding et al., 2009, Biondi et al., 2004, Crabtree et al., 2003). The 4 conventional *Men1*^+/−^ models consist of 1 with deletion of exons 3–8 (Crabtree et al., 2001), 1 with deletion of the transcriptional start site and exon 2 (Biondi et al., 2002, Loffler et al., 2007a, Loffler et al., 2007b), 1 with deletion of exon 3 (Bertolino et al., 2003a, Bertolino et al., 2003b) and 1 with deletion of exons 1 and 2 (Harding et al., 2009). In each of these models pituitary tumours occurred in 26–45% of mice by 18 months of age, with prolactin-expressing tumours being the most common. For each of the conventional models homozygous (*Men1*^−/−^) mice were embryonic lethal. To study the effects of complete loss of menin in specific tissues and organs, 2 conditional models have been generated. In one model, mice with *Men1* exons 3–8 floxed with LoxP were crossed with mice expressing Cre under the control of the rat insulin promoter, which as well as being expressed in pancreatic islets is weakly expressed in the pituitary. This resulted in the occurrence of pancreatic beta cell tumours, and pituitary adenomas which immunostained for prolactin in up to 58% of mice (Crabtree et al., 2003). In another model exon 3 of *Men1* was floxed with LoxP sites and crossing this line with mice expressing *Rip-Cre*, was reported to result only in the development of pancreatic tumours (Bertolino et al., 2003a).

Mouse models that have also been generated for other pituitary adenoma associated human disorders include: MEN4; Carney Complex; Acromegaly/gigantism and FIPA. Thus, *Cdkn1b*^+/−^ mice develop tumours consistent with MEN4, which is characterised by the occurrence of pituitary and parathyroid tumours in association with other endocrine tumours (e.g. of gonads and adrenals) (Kiyokawa et al., 1996, Nakayama et al., 1996, Fero et al., 1998). *Prkar1a*^+/−^ mice develop tumours consistent with Carney Complex syndrome, which is characterised by increased occurrence of different tumour types including myxomas, schwannomas, and endocrine tumours, which include somatotrophinomas, adrenal cortical tumours, sertoli cell tumours, ovarian cysts and thyroid follicular adenomas (Yin et al., 2008). *Cdkn2c*^+/−^ mice develop tumours consistent with acromegaly/gigantism, due to GH secreting tumours (Franklin et al., 1998). The phenotypic features of *Aip*^+/−^ mice are similar to that observed in FIPA patients, who have *AIP* mutations and predominately develop GH secreting adenomas, although some patients may also develop PRL or ACTH secreting and non-functioning adenomas (Leontiou et al., 2008, Vierimaa et al., 2006, Raitila et al., 2010). These models provide valuable resources to study the mechanisms causing pituitary tumours. For example, investigation of the *Aip*^+/−^ mouse model has revealed that the associated pituitary tumours have activation of a hypoxic response, with induction of HIF-1α expression, and that signalling through the HIF-1α binding partners aryl hydrocarbon receptor nuclear translocator (ARNT) and ARNT2 is a key factor in the development of pituitary tumours, after loss of Aip (Raitila et al., 2010).

Studies of other mouse models deleted for genes that were not previously known to be associated with the development of pituitary tumours in man have revealed roles for such genes, which includes *Rb*, *Cdkn2b*, *Drd2*, *Prl* and *Prlr*, in pituitary tumourigenesis. The Rb protein is a tumour suppressor with an integral role in cell cycle progression, controlling the passage from G1 into S phase (Weinberg, 1995) and ∼90% of patients with a heterozygous germline *RB* mutation develop childhood onset retinoblastoma. However, *Rb*^+/−^ mice were found not to develop retinoblastoma, but instead to develop pituitary carcinoma, and therefore provide a valuable model for studying the development of pituitary carcinomas (Jacks et al., 1992, Vooijs et al., 1998, Vooijs et al., 2002). Moreover, use of the FLP-frt system to generate conditional *Rb* knockout mice has revealed that mice null for *Rb* specifically in the pituitary, rapidly develop melanotroph tumours (Vooijs et al., 1998), and showed that Rb loss is an initiating step in carcinogenesis, leading to inappropriate entry into S phase of the cell cycle (Vooijs et al., 1998). In addition, use of the Cre-LoxP system to knockout *Rb* in the mouse pituitary proopiomelanocortin (POMC) cell lineages (corticotrophs and melanotrophs), together with expression of firefly luciferase under the control of the POMC promoter has generated a model allowing real time imaging of tumour growth, using bioluminescence, which allows accurate evaluation of the efficacy novel therapies including the chemotherapeutic action of doxorubicin (Vooijs et al., 2002). Finally, the role of p19, encoded by *Cdkn2b*, as a tumour suppressor in regulating pituitary anterior lobe proliferation has been revealed by a conventional knockout mouse model of *Cdkn2b*, as *Cdkn2b*^−/−^ mice developed multiple tumour types including PRL, GH and FSH secreting pituitary adenomas (Bai et al., 2014).

#### 2.2.2 Over-expression and knockin models

Over-expression models have been generated to investigate the roles: of hormones in pituitary tumourigenesis, e.g. over-expression of the growth hormone releasing hormone (*Ghrh*) gene in mice resulted in pituitary adenomas that led to excessive GH secretion (Asa et al., 1992b); and of oncogenes and tumourigenic viruses. Thus, over-expression of some viral proteins, such as Simian virus 40 (SV40) T antigen, which have been termed ‘oncoviruses’ as infection of tissues can lead to tumour development, generated a mouse model with somatotrophinomas (Stefaneanu et al., 1992), and over-expression of a polyoma early region promoter linked to a cDNA encoding polyoma large T antigen (PyLT), in mice, induced development of ATCH-secreting pituitary tumours, thereby providing a model for Cushing's disease (Helseth et al., 1992). Transgenic over-expression of cortocotropin releasing hormone (*Crh*) in mice has also generated a model of Cushing's disease (Stenzel-Poore et al., 1992), and an ENU induced mouse mutant with a *Crh* promoter mutation has been reported to develop Cushing's Syndrome (Bentley et al., 2014). In addition, use of constructs containing promoters of genes expressed in the pituitary has facilitated generation of targeted models. Thus, mice harbouring a transgene with the SV40 early gene encoding large T antigen ligated to the POMC promoter, developed melanotroph tumours (Low et al., 1993), and mice harbouring the SV40 T antigen targeted to gonoadotroph cells using the follicle stimulating hormone β (Fshβ) promoter, generated a model of non-functioning adenomas (Kumar et al., 1998).

The role of the pituitary transforming gene (*Pttg*) has also been established by the use of tissue-specific transgenic over-expression mouse models (Donangelo et al., 2006, Abbud et al., 2005). *Pttg*, which was first identified from rat pituitary tumour cells (Pei and Melmed, 1997), and subsequently shown to be over-expressed in human pituitary tumours (Saez et al., 1999), encodes a securin protein that plays a role in cell transformation, aneuploidy, apoptosis and tumour microenvironment communication (Vlotides et al., 2007). Mice with targeted pituitary *Pttg* over-expression, driven by the pituitary specific alpha subunit glycoprotein promoter have been reported to develop focal pituitary hyperplasia, whilst mice with *Pttg* inactivation have pituitary hypoplasia (Donangelo et al., 2006), consistent with a pituitary tumourigenic role of *Pttg*.

The tumourigenic role of the High mobility group A (*Hmga*) genes has also been investigated by transgenic over-expression in mouse models. HMGA proteins are architectural transcription factors as they regulate the assembly of complexes important for gene transcription (Fedele et al., 2006). The two HMGA genes, *HMGA1* and *HMGA2*, are expressed at high levels during embryogenesis, and in some human carcinomas, but are not expressed in the majority of adult normal tissues (Fedele et al., 2006). The oncogenic potential of the HMGA genes in pituitary tumourigenesis has been evaluated by the generation of global transgenic over-expression mouse models (Fedele et al., 2005, Fedele et al., 2002). Over-expression of either *Hmga1* or *Hmga2* resulted in the development of mixed somatotroph/lactotroph pituitary adenomas by 16 months of age (Fedele et al., 2005, Fedele et al., 2002), and Hmga2 has been reported to enhance E2F1 activity, which is usually repressed by Rb to prevent progression through the cell cycle (Fedele et al., 2006).

Knockin mouse models of the cyclin dependent kinases (CDKs) and cyclin dependent kinase inhibitors (CKIs) have been reported to develop pituitary tumours (Sotillo et al., 2001, Besson et al., 2006, Roussel-Gervais et al., 2010). The CKIs, p16, p15, p18 and p19, inhibit the activity of the CDKs CDK4 and CDK6, thereby preventing phopsphorylation of Rb, and G1 to S phase transition (Cicenas et al., 2014), and a mouse with a *Cdk4* point mutation (Arg24Cys) that resulted in insensitivity to the CKIs (Sotillo et al., 2001), developed tumours of the pituitary, pancreas and testes (Sotillo et al., 2001). In addition, a mouse with a mutation (Ser10Ala) of *Cdkn1b*, which encodes p27, a member of the p21 family of CKIs that abrogated its ability to bind CDKs (Besson et al., 2006), resulted in the development of pituitary adenomas (Besson et al., 2006). Moreover, one of the target cyclin complexes of p27 is cyclin E-CDK2, and a transgenic over-expression mouse model has indicated that cyclin E expression in the POMC lineage is sufficient to cause cells to re-enter the cell cycle and initiate pituitary adenoma growth (Roussel-Gervais et al., 2010). These mouse models, which highlight the critical role of the G1 checkpoint in pituitary tumourigenesis, provide pre-clinical models for assessing the efficacy of small molecule inhibitors of cyclin dependent kinases that are being developed for the treatment of cancers (Cicenas et al., 2014).

#### 2.2.3 Double mutant models

Studies of double mutant mouse models have provided important *in vivo* mechanistic insights into cell cycle regulation during pituitary tumour development, and the progression of benign pituitary adenomas to carcinomas. Thus, 12 double mutant mouse models comprising: *Pttg*/*Rb*^+/−^; *Rb*^+/−^/*Ini1*^+/−^; *Rb*^+/−^*/Arf*^−/−^; *Rb*^+/−^/*p53*^−/−^; *Cdk4*^*R*/*R*^/*Cdkn1b*^−/−^; CyclinE/p27^−/−^; p18^−/−^/p27^−/−^; p18^−/−^/αSU^−/−^; Ink4c^−/−^/Arf^−/−^; *Men1*^+/−^*/Rb*^+/−^; *Men1*^+/−^/*Cdk2*^−/−^ and *Men1*^+/−^/*Cdk4*^−/−^, have been generated (Table 2). Five of these double mutant mouse models utilised *Rb*^+/−^ mice to investigate co-operative pathways in pituitary tumourigenesis, and revealed that: over-expression of the pituitary hyperplasia-promoting gene *Pttg* in combination with *Rb*^+/−^ increases the volume and prevalence of anterior pituitary tumours, whereas loss of *Pttg* expression, as occurring in *Pttg*^−/−^ mice can protect against tumours initiated by loss of Rb (Donangelo et al., 2006, Chesnokova et al., 2005); Rb is epistatic to a member of the SW1/SNF chromatin remodelling complex, Ini1, in tumour suppression (Guidi et al., 2006); loss of the tumour suppressor proteins p53 or Arf (that stabilises p53 protein), as in occurring in p53^−/−^ or Arf^−/−^ mice, in combination with Rb^+/−^ accelerated pituitary tumour development (Harvey et al., 1995, Tsai et al., 2002); and *Men1*^+/−^/*Rb*^+/−^ mice did not have significant differences in the occurrence or age of onset of pituitary, or other tumours, when compared to *Men1*^+/−^ or *Rb*^+/−^ mice (Loffler et al., 2007a, Loffler et al., 2007b), thereby indicating that menin and Rb do not have an additive effect and are therefore likely to function in a common pathway to suppress tumour development. The remaining 7 double mutant models have utilised loss of CKIs from both the INK4 and p21 family to investigate the interaction between different cyclins, CDK, CKIs and their substrates (Rb) in the pituitary (Franklin et al., 1998, Roussel-Gervais et al., 2010, Sotillo et al., 2005, Zindy et al., 2003, Lloyd et al., 2002). Thus, *Cdk4*^*R*/*R*^/*Cdkn1b*^+/−^ and *Cdk4*^*R*/*R*^/*Cdkn1b*^−/−^ mice, which would be null for p27 and have a mutant of CDK4 that renders it insensitive to INK4 inhibitors, have been reported to develop pituitary tumours with complete penetrance and a short latency, thereby indicating that there is co-operativity between p27 and CDK4 (Sotillo et al., 2005). Such co-operativity was not identified to occur between p18 and CDK4 in similar studies using the *Cdk4*^*R*/*R*^*/Cdkn2c*^−/−^ mouse model (Sotillo et al., 2005). These results indicate that p27 may have a wider function that just inhibiting CDK4 activity, which has previously been considered to be the predominant function of p18. Moreover, it has also been reported that up-regulation of cyclin E (using a transgene construct under the control of the POMC promoter, *Tg*-*PCE*) and knockout of p27 (*Cdkn1b*^−/−^) in the *Tg*-*PCE*/*Cdkn1b*^−/−^mouse model, leads to increased pituitary adenoma incidence and frequency (Roussel-Gervais et al., 2010) and that p27 and p18 mediate two separate pathways to collaboratively suppress tumourigenesis (Franklin et al., 1998). *Men1*^+/−^/*Cdk4*^−/−^ mice have been demonstrated to not develop any tumours, whereas *Men1*^+/−^/*Cdk2*^*−/−*^ mice develop pituitary and pancreatic tumours comparable to those in *Men1*^+/−^ only mice. (Gillam et al., 2015). This indicates that Cdk4 activity is important for *Men1*-associated tumourigenesis to occur, and that menin may predominantly suppress cell cycle progression through the INK4-Cdk4/6-cyclin D-pRb pathway. Overall, these studies of mutant mouse models with targeted disruption of the cell cycle regulating proteins have provided mechanistic insights in pituitary tumourigenesis.

### 2.3 Rat pituitary neoplasia models

Some rat strains, including Sprague Dawley rats, are more prone to pituitary tumour development (Son and Gopinath, 2004, Son et al., 2010, Carlus et al., 2013). For example, a variant of the MEN syndromes, termed MENX, was discovered to occur spontaneously in Spague–Dawley rats, which developed parathyroid adenomas, pancreatic islet cell hyperplasia, thyroid c-cell hyperplasia, bilateral phaeochromocytomas and paragangliomas (Marinoni et al., 2013). MENX was inherited as an autosomal recessive disorder and the rats were found to have a germline homozygous frameshifting insertion mutation in the *Cdkn1b* gene (Fritz et al., 2002). Studies in patients with MEN1-like tumours, but without *MEN1* mutations, revealed some to have *CDKN1B* mutations. To date 10 different MEN4-associated mutations of *CDKN1B* have been reported, and the MEN4 patients have parathyroid tumours in association with pituitary adenomas and other tumours of the gonads, adrenals, thyroid and kidney (Thakker, 2013, Pardi et al., 2014).

Rat models of pituitary neoplasia have also been generated by implanting tumour cells or using drugs. Thus, a rat model for Cushing's syndrome was generated by implanting a medullary thyroid carcinoma cell line, which stably over-expressed CRH, into WAG/Rij rats (Asa et al., 1992a). Exposure of these rats to elevations in circulating CRH, increased anterior pituitary cell proliferation and circulating ACTH levels (Asa et al., 1992a, Asa et al., 1992b). In addition, a rat model for lactotrophinomas was generated by long-term administration of oestrogens to the F344 and Holtzmon strains (Wiklund et al., 1981a, Wiklund et al., 1981b). Such susceptibility to oestrogen treatment in inducing pituitary tumours in rats varies between strains. For example, oestrogen treatment has been shown to differ between strains, with F344 female rats the most susceptible (Mitsui et al., 2013, Wiklund et al., 1981a, Wiklund et al., 1981b), and with Wistar-Kyoto rats not developing pituitary tumours upon oestrogen treatment due to lack of lactotroph cell proliferation (Mitsui et al., 2013). Oestrogens are known to stimulate expression of IGF-1, which leads to increased proliferation. However in Wistar-Kyoto rats the response to IGF-1 was reduced, with IGF-1 target gene expression (*Wnt4*, *Stc1*, *Mybl1* and *Myc*) attenuated or abolished (Mitsui et al., 2013), and this may explain the lack of lactotroph cell proliferation following oestrogen treatment in Wistar-Kyoto rats.

### 2.4 Zebrafish pituitary neoplasia model

A zebrafish model that develops corticotroph adenomas has been generated by utilising transgenic expression of the *Pttg* gene that is targeted specifically to the pituitary POMC lineages (Liu et al., 2011). This study illustrates that zebrafish models for pituitary tumours can be generated, and opens the way to utilising additional methods for the study of pituitary tumourigenesis. Thus, zebrafish knockout models for pituitary tumours could be generated by using morpholino oligomers, which are antisense oligomers that bind complementary messenger RNAs thereby blocking gene expression and leading to gene knockdown (Angotzi et al., 2011). In addition, ENU mutagenesis can be used to generate hypermorphic or hypomorphic phenotypes in zebrafish (Solnica-Krezel et al., 1994). Studies in zebrafish offer many advantages, such as a high throughput platform for assessing gene abnormalities and for drug efficacy studies, as demonstrated by a study that evaluated the use of the cell cycle inhibitor R-roscovitine for the treatment of corticotroph tumours, the results of which were subsequently confirmed in a mouse model (Liu et al., 2011).

## 3 Use of the Men1^+/−^ mouse for evaluating novel therapies

Mouse models for pituitary tumours have provided pre-clinical models for evaluating new therapies for pituitary tumours. This is illustrated by the use of the *Men1* mouse model, which has been used to assess the role of *Men1* gene replacement therapy and a monoclonal antibody to the vascular endothelial growth factor (VEGF) (Walls et al., 2012, Korsisaari et al., 2008). The tumour suppressor role of menin, encoded by *Men1*, suggested that restoration of menin expression in tumours would reduce proliferation. Indeed, gene replacement therapy using an adenoviral vector containing *Men1* cDNA that was delivered directly to the pituitary tumours of *Men1*^+/−^ mice, restored menin expression in the pituitary tumours and reduced their proliferation, without significant adverse effects or increased mortality (Walls et al., 2012). These findings indicate that viral delivery of *MEN1* may be a potential treatment for pituitary and other neuroendocrine tumours associated with MEN1 mutations. Pituitary tumours, in common with other neoplasms, have increased angiogenesis, and angiogenic pathways that can be targeted by monoclonal antibodies to VEGF. Indeed, the monoclonal antibody G6-31 has been reported to inhibit VEGF-induced pituitary tumour growth in conventional *Men1*^+/−^ mice (Korsisaari et al., 2008). Such mouse models therefore provide an important resource for the pre-clinical evaluation of novel therapies for pituitary tumours.

## 4 Conclusion

Studies of animal models of pituitary neoplasias have helped to advance our knowledge about the biological mechanisms of pituitary tumourigenesis. Thus, these models, predominately in mice, which have been generated by a number of approaches including gene knockout and over-expression of genes, have provided novel insights into the regulation of the cell cycle and its dysfunction in pituitary tumourigenesis. In addition, they provide pre-clinical models for the evaluation of novel and emerging therapies.

## Figures and Tables

**Table 1:** Genetic abnormalities identified from human studies to be associated with pituitary neoplasias.

Gene	Tumour type/Syndrome	Gene defect	Reference
*AIP*	FIPA	Germline inactivating mutation	(Beckers et al., 2013)
Young onset sporadic pituitary macroadenomas
*BMP-4*	Corticotrophinomas	Gene down-regulation	(Giacomini et al., 2006)
Somatotrophinomas	Gene over-expression
Prolactinomas	Gene over-expression
*CASP8*	Functioning and non-functioning adenomas	Methylation mediated gene silencing	(Bello et al., 2006)
*CCNA2*	MEN1 patients without *MEN1*, *CASR* or *HRPT2* mutations	Gene over-expression	(Agarwal et al., 2009)
*CCNB1*	MEN1 patients without *MEN1*, *CASR* or *HRPT2* mutations	Gene over-expression	(Agarwal et al., 2009)
*CCNB2*	MEN1 patients without *MEN1*, *CASR* or *HRPT2* mutations	Gene over-expression	(Agarwal et al., 2009)
*CCND1*	Non-functioning adenomas	Gene over-expression	(Jordan et al., 2000)
*CCNE1*	Cushing's syndrome	Gene over-expression	(Jordan et al., 2000, Jaffrain-Rea et al., 2013)
MEN1 patients without *MEN1*, *CASR* or *HRPT2* mutations
*CDH1*	Somatotrophinomas with prominent fibrous bodies	Methylation-mediated gene silencing	(Zhou et al., 2013)
*CDH13*	Functioning and non-functioning adenomas	Methylation-mediated gene silencing, correlating with tumour aggressiveness	(Qian et al., 2007)
*CDKN1A*	Functioning and non-functioning adenomas	Gene down-regulation	(Hiyama et al., 2002)
*CDKN1B*	MEN4 patients	Germline inactivating mutation	(Pellegata et al., 2006)
*CDKN2A*	Functioning and non-functioning adenomas	Methylation-mediated gene silencing	(Zhou et al., 2013)
*CDKN2B*	Functioning and non-functioning adenomas	Methylation-mediated gene silencing	(Zhou et al., 2013)
*CDKN2C*	Functioning and non-functioning adenomas	Methylation-mediated gene silencing	(Zhou et al., 2013)
*DAPK* family	Functioning and non-functioning adenomas	Loss of expression	(Simpson et al., 2002)
*FGFR2*	Functioning adenomas	Methylation-mediated gene silencing	(Zhu et al., 2007)
*FGFR4*	Functioning adenomas	Constitutively phosphorylated	(Ezzat et al., 2002)
*GADD45B*	Gonadotrophinoma	Gene silencing	(Michaelis et al., 2011)
*GADD45G*	Functioning, but more commonly in non-functioning adenomas	Gene silencing	(Zhou et al., 2013)
*GNAS*	Somatotrophinomas	Mutations detected	(Mantovani et al., 2010)
*HMGA*-1	Prolactinomas	Gene over-expression	(De Martino et al., 2009)
*HMGA*-2	Prolactinomas	Gene over-expression	(Fedele et al., 2006)
*LGALS3*	Lactotrophinomas	Gene over-expression	(Righi et al., 2010)
Corticotrophinomas
*MEG3*	Non-functioning adenomas	Methylation-mediated gene silencing	(Zhang et al., 2010)
*MEN1*	MEN1	Inactivating mutations and gene deletions	(Thakker, 2010)
Young onset sporadic pituitary adenomas
*MGMT*	Carcinomas	Methylation-mediated gene silencing	(Zhou et al., 2013)
*PLAGL1*	Non-functioning adenomas	Methylation-mediated gene silencing	(Pagotto et al., 2000)
*PRKAR1A*	Somatotrophinomas	Gene down-regulation	(Kirschner, 2010)
Non-functioning adenomas
*PTAG*	Adenomas (subtype not defined)	Methylation-mediated gene silencing	(Bahar et al., 2004)
*PTTG1*	Functioning and non-functioning adenomas	Gene over-expression	(Salehi et al., 2008)
*RAS* family	Functioning and non-functioning adenomas	Activating mutations	(Karga et al., 1992)
*RASSF1*	Functioning and non-functioning adenomas	Methylation-mediated gene silencing	(Qian et al., 2005)
*RASSF3*	Somatotrophinomas	Methylation-mediated gene silencing	(Peng et al., 2013)
*RB1*	Aggressive adenomas	Rare inactivating mutations, methylation-mediated gene silencing	(Pei et al., 1995)
Carcinoma
*SOCS1*	Somatotrophinomas	Methylation-mediated gene silencing	(Buslei et al., 2006)
Corticotrophinomas
Non-functioning adenomas
*SOX2*	Early onset pituitary adenomas	Rare gene deletion	(Alatzoglou et al., 2011)
*TP53*	Carcinoma	Rare inactivating mutations	(Tanizaki et al., 2007) (Kawashima et al., 2009)
Atypical corticotrophinoma	Rare inactivating mutation in one patient
*USP8*	Corticotroph adenomas	Dominant gain of function mutations	(Reincke et al., 2015, Jian et al., 2015)

**Table 2:** Mouse models of pituitary neoplasia.

Tumour type	Tumour site	Syndrome/Disorder	Mouse model (gene – method)	Gender phenotype	Reference
**Prolactinoma** (secreting prolactin)	Isolated	Carney Complex	***Prkar1a*** – Tissue-specific homozygous knockout	__	(Yin et al., 2008)
Acromegaly/ Gigantism	***Ghrh*** – Transgenic over-expression	__	(Asa et al., 1992a, Asa et al., 1992b)
Non-syndromic	***Prl*** – Heterozygous and homozygous knockout	Female only	(Cruz-Soto et al., 2002)
***Prlr*** – Homozygous knockout	Male and female	(Schuff et al., 2002)
***TGFα*** - Tissue-specific transgenic Over-expression	Females only	(McAndrew et al., 1995)
*ptd*-***FGFR4*** – Tissue-specific transgenic Over-expression	Male and female	(Ezzat et al., 2002)
***Prop1*** – Tissue specific transgenic Over-expression	Female only	(Egashira et al., 2008, Cushman et al., 2001)
***Drd2*** – Homozygous knockout	Females only	(Kelly et al., 1997, Asa et al., 1999)
***Cyclin E*** – Tissue-specific transgenic over-expression	__	(Roussel-Gervais et al., 2010)
Multiple	MEN1	***Men1*** – Heterozygous knockout	Higher incidence in females	(Crabtree et al., 2001, Bertolino et al., 2003a, Bertolino et al., 2003b, Biondi et al., 2002, Loffler et al., 2007a, Loffler et al., 2007b, Harding et al., 2009, Lemos et al., 2009)
***Men1*** – Tissue-specific homozygous knockout	Higher incidence in females	(Biondi et al., 2004, Crabtree et al., 2003)
Non-syndromic	***Pttg***:***Rb*** – Tissue-specific transgenic *Pttg* over-expression: Heterozygous ***Rb*** knockout	Male and female *NB Pttg* knockout x *Rb* knockout is protective for pituitary adenomas	(Donangelo et al., 2006, Chesnokova et al., 2005)
**p19** – Homozygous knockout	Male and female	(Bai et al., 2014)
***Hmga1*** – Transgenic over- expression	Higher incidence in females	(Fedele et al., 2005)
***Hmga2*** – Transgenic over- expression	Higher incidence in females	(Fedele et al., 2002)
***AVP***-**SV40 large T antigen** – Transgenic expression	__	(Stefaneanu et al., 1992)
**Gonadotrophinoma** (secreting follicle stimulating hormone, lutenising hormone, or non-functioning)	Isolated	Non-syndromic	***FSHβ***-**SV40 temperature sensitive T antigen** –Transgenic expression	Male only	(Kumar et al., 1998)
**Pttg** – Tissue-specific transgenic over-expression	Male and Female	(Donangelo et al., 2006, Abbud et al., 2005)
***Prop1*** – Tissue-specific transgenic Over-expression	Female only	(Egashira et al., 2008, Cushman et al., 2001)
***Cyclin E*** – Tissue-specific transgenic over-expression	__	(Roussel-Gervais et al., 2010)
Multiple	MEN1	***Men1*** – Heterozygous knockout	Higher incidence in females	(Crabtree et al., 2001, Bertolino et al., 2003a, Bertolino et al., 2003b, Biondi et al., 2002, Loffler et al., 2007a, Loffler et al., 2007b, Harding et al., 2009, Lemos et al., 2009)
Non-syndromic	***Pttg***:***Rb*** – Tissue-specific transgenic *Pttg* over-expression: Heterozygous *Rb* knockout	Male and female *NB Pttg* knockout x *Rb* knockout is protective for pituitary adenomas	(Donangelo et al., 2006, Chesnokova et al., 2005)
**p19** – Homozygous knockout	Male and female	(Bai et al., 2014)
**Somatotrophinoma** (secreting growth hormone)	Isolated	FIPA	***Aip*** – Heterozygous knockout	Male and female	(Raitila et al., 2010)
Carney Complex	***Prkar1a*** – Tissue-specific homozygous knockout	__	(Yin et al., 2008)
Acromegaly/ Gigantism	***Ghrh*** – Transgenic over- expression	__	(Asa et al., 1992a, Asa et al., 1992b)
Non-syndromic	***Prop1*** – Tissue-specific transgenic over-expression	Female only	(Egashira et al., 2008, Cushman et al., 2001)
***Cyclin E*** – Tissue-specific transgenic over-expression	__	(Roussel-Gervais et al., 2010)
Multiple	MEN1	***Men1*** – Heterozygous knockout	Higher incidence in females	(Crabtree et al., 2001, Bertolino et al., 2003a, Bertolino et al., 2003b, Biondi et al., 2002, Loffler et al., 2007a, Loffler et al., 2007b, Harding et al., 2009, Lemos et al., 2009)
Non-syndromic	***Pttg***:***Rb*** – Tissue-specific transgenic *Pttg* over-expression: Heterozygous *Rb* knockout	Male and female *NB Pttg* knockout x *Rb* knockout is protective for pituitary adenomas	(Donangelo et al., 2006, Chesnokova et al., 2005)
**p19** – Homozygous knockout	Male and female	(Bai et al., 2014)
***Hmga1*** – Transgenic over- expression	Higher incidence in females	(Fedele et al., 2005)
***Hmga2*** – Transgenic over- expression	Higher incidence in females	(Fedele et al., 2002)
***AVP***-**SV40 large T antigen** – Transgenic over-expression	__	(Stefaneanu et al., 1992)
**Corticotrophinoma** (secreting adrenocorticotropic hormone)	Isolated	Acromegaly/ Gigantism	**p18** – Homozygous knockout	Male and female	(Franklin et al., 1998)
**p18**:**p27** – Homozygous p18 knockout: Homozygous p27 knockout	Male and female Accelerated rate of adenomas	(Franklin et al., 1998)
Cushing's	***PyLT*** – Transgenic over- expression	Male and female	(Helseth et al., 1992)
***Crh*** – Transgenic over- expression	Male and female	(Stenzel-Poore et al., 1992)
***Crh*** – Heterozygous gain-of-function mutation (ENU)	Male and female	(Bentley et al., 2014)
Non-syndromic	***Pttg***:***Rb*** – Tissue-specific transgenic *Pttg* over-expression: Heterozygous *Rb* knockout	Male and female *NB Pttg* knockout x *Rb* knockout is protective for pituitary adenomas	(Donangelo et al., 2006, Chesnokova et al., 2005)
Multiple	Non-syndromic	***Rb***:***Ini1*** – Heterozygous *Rb* knockout: *Heterozygous* *Ini1* knockout	__ Accelerated carcinoma development	(Guidi et al., 2006)
**SV40 large T antigen** –Transgenic expression under *Pomc* promoter	__	(Low et al., 1993)
***AVP***-**SV40 large T antigen** – Transgenic over- expression	__	(Stefaneanu et al., 1992)
**Thyrotrophinoma** (secreting thyroid stimulating hormone)	Isolated	Carney Complex	***Prkar1a*** – Tissue-specific homozygous knockout	__	(Yin et al., 2008)
Acromegaly/ Gigantism	***Ghrh*** – Transgenic over- expression	__	(Asa et al., 1992a, Asa et al., 1992b)
Non-syndromic	***p18***:*α****SU*** – Homozygous p18 knockout: Homozygous *αSU* knockout	__	(Lloyd et al., 2002)
***Prop1*** – Tissue-specific transgenic over-expression	Female only	(Egashira et al., 2008, Cushman et al., 2001)
Multiple	Non-syndromic	***AVP***-**SV40 large T antigen** – Transgenic over- expression	__	(Stefaneanu et al., 1992)
**Craniopharyngiomas**	Isolated	Non-syndromic	***Ctnnb1*** – Tissue-specific knockout of exon 3, rendering *Ctnnb1* degradation resistant	__	(Gaston-Massuet et al., 2011)
**Undefined** adenoma subtype	Isolated	Non-syndromic	***Bmi1*** – Tissue-specific transgenic over-expression	__	(Westerman et al., 2012)
Multiple	MEN4	***Cdkn1b*** – Homozygous knockout	Male and female - females infertile	(Kiyokawa et al., 1996, Nakayama et al., 1996, Fero et al., 1996)
***Cdkn1b*** – Homozygou*s* inactivating mutation knockin	__	(Besson et al., 2006)
Non-syndromic	***Cdk4*** – mutation (rendering protein insensitive to INK4 inhibitors) knockin	Male and female	(Sotillo et al., 2001)
***Cdk4***:***Cdkn1b*** – Transgenic *Cdk4* mutation knockin: Homozygous *Cdkn1b* knockout	__	(Sotillo et al., 2005)
***Men1***:***Cdk2*** – Heterozygous *Men1* knockout: homozygous *Cdk2* knockout	Male and female	(Gillam et al., 2015)
**Cyclin E**:**p27** – Tissue-specific transgenic Cyclin E over-expression: Homozygous p27 knockout	__ Increased adenoma size, proliferation and frequency	(Roussel-Gervais et al., 2010)
***Ink4c***:***Arf*** – Homozygous *Ink4c* knockout: Homozygous *Arf* knockout	Male and female	(Zindy et al., 2003)
***Rb*** – Heterozygous knockout	__	(Jacks et al., 1992)
***Rb*** – Tissue-specific homozygous knockout	__	(Vooijs et al., 1998, Vooijs et al., 2002)
***Rb***:***Arf*** – Heterozygous *Rb* knockout: Homozygous *Arf* knockout	__ Accelerated tumour development	(Tsai et al., 2002)
***Rb***:**p53** – Heterozygous *Rb* knockout: Heterozygous and homozygous p53 knockout	__	(Harvey et al., 1995)

- = not defined; MEN1 – multiple endocrine neoplasia type 1; MEN4 - multiple endocrine neoplasia type 4; FIPA – familial isolated pituitary adenomas.
